# Legionnaires' disease on azithromycin leading to lofty liver levels

**DOI:** 10.1016/j.idcr.2025.e02221

**Published:** 2025-04-04

**Authors:** Sagar Modh, Mark Grijalva, Vignesh Krishnan, Angel Chacko, Shraboni Dey, Paranjyothy Rao Pirangi Sanjeeva, Adam Atoot

**Affiliations:** a School of Medicine, Xiamen University, Fujian, China; b HMH Palisades Medical Center, 7600 River road, NJ, USA

**Keywords:** *Legionella* pneumonia, Drug-induced liver injury, Macrolide antibiotic-induced DILI, Fluoroquinolone antibiotic-induced DILI, R factor for liver injury

## Abstract

Legionnaires' disease is a serious, life-threatening pneumonia caused by the bacterium *Legionella pneumophila*. *Legionella* is a gram-negative bacillus transmitted via inhalation of water droplets, usually from air conditioners or hot tubs. Unlike many respiratory pathogens, *Legionella* does not spread from person to person. This bacterium primarily affects the pulmonary system, causing atypical community-acquired pneumonia often associated with gastrointestinal symptoms such as diarrhea. In rare cases, *Legionella* can involve the liver, leading to acute hepatitis. Fluoroquinolones and macrolides are the most effective and commonly used antibiotics for treating Legionnaires' disease. However, both antibiotic classes carry a potential risk of hepatotoxicity, which can result in elevated liver enzymes. Additionally, *Legionella*-induced liver injury may increase susceptibility to DILI. Here, we present a case of Legionnaires' disease treated with azithromycin, complicated by elevated liver enzymes, highlighting the need for careful monitoring of liver function during treatment.

**Key Clinical Message**

Legionnaires' disease can present with atypical symptoms of acute hepatitis, even when liver function tests are initially normal. Macrolide antibiotics, commonly used to treat Legionnaires' disease, can cause drug-induced liver injury (DILI). Patients with *Legionella* infection may experience mild liver injury with normal or near-normal liver function tests, which may predispose them to DILI from azithromycin. Clinicians should remain vigilant for this potential complication and consider alternative antibiotics if liver function tests worsen during therapy.

## Introduction

*Legionella pneumophila* is a Gram-negative bacterium that can cause severe illness in humans and is most commonly associated with respiratory manifestations. Rarely, this organism can also present in extrapulmonary forms such as acute hepatitis. However, specific incidence rates of Legionella-induced liver injury are not well-documented in the available literature. Most reports consist of individual case studies or small series, making it difficult to establish a precise rate. This case report explores a rare presentation of *Legionella* infection in a patient receiving azithromycin, leading to hepatic injury.

*Legionella* is a leading cause of reported waterborne disease outbreaks in the United States. This bacterium can also cause a milder illness known as Pontiac fever, characterized by flu-like symptoms. However, *Legionella* most commonly causes community-acquired atypical pneumonia. Transmission occurs through inhalation or aspiration of aerosolized organisms, most commonly from hot tubs and air conditioners. Symptoms include high fever, chills, cough (sometimes productive), shortness of breath, muscle aches, headaches, and gastrointestinal issues. *Legionella* is responsible for 57 % of outbreaks of waterborne infections [1], [2]. Approximately 10,000–50,000 cases of *Legionella* occur annually in the United States, with fatality rates ranging from 30 % to 50 % if treatment is delayed [3], [4].

Drug-induced liver injury (DILI) is considered the most common cause of acute liver failure [16]. Azithromycin and levofloxacin are commonly used antibiotics for empirically treating community-acquired pneumonia, including *Legionella pneumophila*. However, these antibiotics pose a challenge due to their potential hepatotoxic effects, which can further compromise liver function, particularly in patients with *Legionella*-induced acute hepatitis. Azithromycin-induced liver injury is rare, with transient, asymptomatic liver enzyme elevations occurring in up to 1–2 % of patients during acute treatment, as reported in large clinical trials. These elevations are typically self-limiting and occur at similar rates in comparator groups, making clinically significant DILI much rarer. However, due to its widespread use, azithromycin remains a notable contributor to overall DILI cases [14]. Additionally, a large retrospective study conducted in the U.S. in 2023 estimated the incidence of clinically significant azithromycin-associated DILI at 4.36 cases per 10,000 exposed individuals (95 % CI: 2.93–6.49) [15].

The case presented here contributes to the understanding of *Legionella’s* diverse presentations. This report describes a case of *Legionella* infection treated with azithromycin, leading to elevated liver enzymes. It highlights the importance of including *Legionella* in the differential diagnosis for acute liver dysfunction, even in the absence of a recent respiratory illness.

## Case history/examination

We present the case of a 35-year-old female with a history of type II diabetes mellitus who presented with shortness of breath and associated fevers. On arrival, she appeared ill, was febrile to 101.3 °F (38 °C), tachycardic to 130 bpm, and was saturating at 95 % on 5 L nasal cannula. She reported taking acetaminophen for the past few days to manage her fever. Her routine medications included metformin XR 850 mg once daily for diabetes. She denied the use of any herbal remedies, smoking, or alcohol intake.

## Methods

Laboratory results were notable for leukocytosis of 18.6 × 10 ³/µL with a neutrophilic predominance of 90.5 %. Hyponatremia was present with a sodium level of 132 mmol/L. Creatinine was 1.05 mg/dL (without a known baseline) (normal: 0.7–1.3 mg/dL). Liver function tests showed an alkaline phosphatase level of 110 U/L (normal: 46–120 U/L), aspartate aminotransferase (AST) of 41 U/L (normal: 8–33 U/L), and alanine aminotransferase (ALT) of 49 U/L (normal: 14–40 U/L). Total bilirubin was 0.4 mg/dL (normal: 0.1–1.2 mg/dL). Coagulation studies revealed a prothrombin time of 16.6 s (normal: 11–13.5 s) and a partial thromboplastin time of 45.2 s (normal: 25–35 s).

**Fig. 1:**
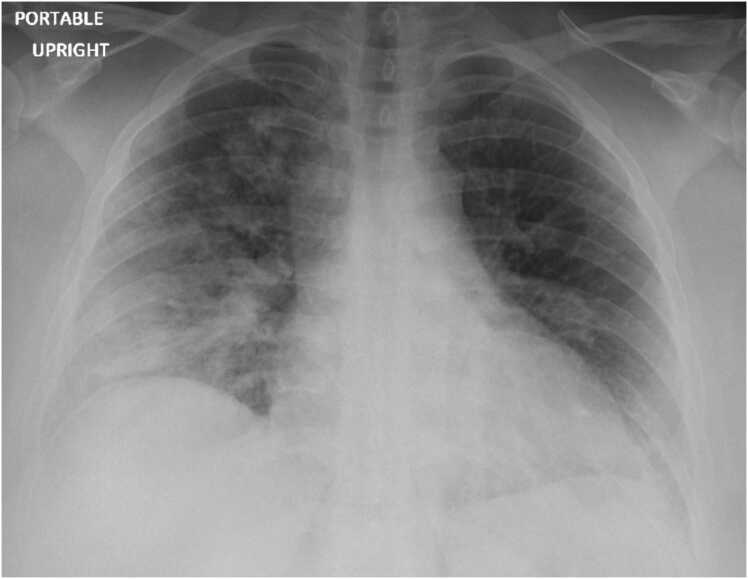
Chest radiograph.

The initial chest radiograph (Fig. 1) showed mild pulmonary vascular congestion with hazy airspace opacities occupying much of the right lateral and lower lung, as well as the left lung base. Empiric treatment for community-acquired pneumonia was initiated with ceftriaxone 1 g once daily and azithromycin 500 mg once daily. A chest CT scan revealed bilateral airspace disease, suggestive of multifocal pneumonia. Routine blood and sputum cultures were collected, and urine antigen tests for *Streptococcus* and *Legionella* were ordered. The *Legionella* urinary antigen test returned positive, leading to the discontinuation of ceftriaxone while azithromycin was continued.

Upon further questioning, the patient reported staying at an Airbnb, where she had shared a hot tub with other people. To her knowledge, none of the other individuals who shared the hot tub or the Airbnb had exhibited similar symptoms.

The next day, after discontinuing ceftriaxone and continuing azithromycin (500 mg once daily), the patient experienced a ten-fold increase in serum liver enzymes. AST increased to 444 U/L (normal: 8–33 U/L), and ALT increased to 479 U/L (normal: 14–40 U/L). Alkaline phosphatase was 115 U/L (normal: 46–120 U/L), and total bilirubin was 0.2 mg/dL (normal: 0.2–1.0 mg/dL). The R factor for liver injury was calculated as 12.5.

A hepatitis panel was negative, and the acetaminophen level was < 10 µg/mL. An autoimmune workup, including antinuclear antibody (ANA), anti-smooth muscle antibody, and anti-liver kidney microsomal-1 antibody, was performed, all of which were within normal limits. Acute viral hepatitis was ruled out, as the patient tested negative for hepatitis A IgM, HBsAb, HBcAb, and HCV Ab, indicating no evidence of recent or active viral hepatitis infection.

## Conclusion and results

After completing the azithromycin course, the liver-associated enzymes began to trend downward. A liver biopsy was initially considered; however, the patient ultimately declined the procedure. By day six of hospitalization, her liver enzyme levels had started to improve, and she was discharged home with instructions to complete her medication course and follow up with her primary care provider. The following table, (Table 1) Hospital timeline summarizes the timeline of events, including antibiotic administration, liver enzyme trends and other laboratory results.

**Table 1:** Hospital timeline.

Hospital day	Azithromycin	Ceftriaxone	ALT U/L	ALPU/L	ASTU/L	PT/INR/PTT	Total Bilirubin
1	+	+	49*	110	41*	16.6s*/1.46*/45.2*s	0.4
2	+	-	479*	115	444*	NA	0.2
3	+	-	540*	131*	419*	NA	0.3
4	+	-	498*	130*	207*	12.5s/1.10/NA	0.3
5	+	-	559*	128*	180*	12.8s/1.13*/NA	0.4
6	-	-	409*	107	70*	13s/1.15*/ 35.0s	0.4

‘+’ indicates that the patient was receiving the respective medication on the corresponding hospital day.

“-” indicates that the medication was discontinued.

‘*’ denotes an abnormal laboratory value.

NA indicates data not available.

Reference Ranges:

ALT: 14–40 U/L.

ALP: 46–120 U/L.

AST: 8–33 U/L.

PT/INR/PTT: 11–13 s / 0.8–1.10 / 25–35 s.

Total Bilirubin: 0.2–1.0 mg/dL.

## Discussion

This case describes Legionnaires' disease presenting with acute hepatitis. *Legionella pneumophila* is a common cause of community-acquired atypical pneumonia, transmitted through the inhalation of aerosolized water droplets. In this case, the patient likely contracted the bacteria at a ski resort, where she had repeatedly used saunas and hot tubs one week before symptom onset. Notably, her close contacts did not exhibit similar symptoms.

The diagnostic test for Legionnaires' disease is a urine antigen test, which returned positive for our patient on hospital day two. The standard treatment regimen for community-acquired pneumonia includes a combination of beta-lactams and macrolides. Upon admission, the patient was started on azithromycin and ceftriaxone. However, following the positive *Legionella* antigen test, ceftriaxone was discontinued after a single dose, and azithromycin was continued from day two onward. Subsequently, her ALT level increased significantly from 49 U/L to 479 U/L by hospital day two.

The abnormal coagulation findings at admission were most likely due to the acute phase reaction associated with the infection rather than primary liver injury, given the initially normal liver enzyme levels (ALT and AST).

It is difficult to determine whether the liver injury resulted from azithromycin alone or in synergy with *Legionella pneumophila*. Based on the course of events, the macrolide antibiotic was likely a contributing factor. However, without a liver biopsy, the exact etiology cannot be confirmed with full confidence. Given this uncertainty, an objective assessment was performed to evaluate the likelihood of azithromycin-induced liver injury. The Naranjo Adverse Drug Reaction Probability Scale yielded a score of 6, indicating a probable causal relationship. Additionally, the Roussel Uclaf Causality Assessment Method (RUCAM) score was 10, classifying the causality as highly probable. These assessments, along with the sequence of events, improvement in liver enzymes following drug discontinuation, and exclusion of alternative causes, strongly suggest that azithromycin contributed to the patient’s liver dysfunction.

In some cases, *Legionella* can cause mild acute liver injury without significant elevation in liver enzymes, potentially predisposing patients to drug-induced liver injury, which may have played a role in our case.

While initiating antibiotic therapy—and thereby killing the organism—could theoretically lead to liver injury, this is unlikely. Macrolides function by inhibiting the bacterial 50S ribosomal subunit, making them primarily bacteriostatic rather than bactericidal. As a result, they are unlikely to cause drug-induced liver injury through direct bacterial cell lysis or the release of bacterial contents [13].

In our case, based on recommendations from infectious disease and gastroenterology specialists, azithromycin was continued for the full duration of antimicrobial therapy. This led to persistently elevated liver enzymes throughout the six-day hospital stay. Azithromycin was completed on hospital day five, and by day six, ALT levels had decreased from 559 U/L to 409 U/L. This decline suggests that the elevated ALT levels were likely due to azithromycin, as the patient had already been exposed to *Legionella* in the community before starting azithromycin in the hospital.

Total bilirubin levels remained within the normal range throughout hospitalization, possibly due to the liver’s ability to compensate for the injury. The significant rise in ALT and AST, with only a minor increase in ALP, suggests a primarily hepatocellular pattern of liver injury. This pattern rules out ceftriaxone as the cause, as it is known to induce cholestatic liver injury [6], [17].

Moreover, the R factor for liver injury was determined using the formula: R = (ALT ÷ ALT upper limit of normal) ÷ (ALP ÷ ALP upper limit of normal) = (479 ÷ 40) ÷ (115 ÷ 120), resulting in a value of 12.5. An R factor of 12.5 strongly suggests a hepatocellular pattern of injury rather than a cholestatic one.

The overlap of symptoms between COVID-19 and *Legionella* makes it imperative to routinely test for *Legionella* in this post-COVID era [5]. Other laboratory abnormalities associated with *Legionella* include hyponatremia, hypophosphatemia, leukocytosis, elevated ESR and CRP, and, in some cases, myoglobinuria.

The medical literature describes a case report of a patient with *Legionella* infection who developed presumed fluoroquinolone-induced DILI. This patient was started on levofloxacin on hospital day two, which led to drug-induced hepatic injury and elevated liver enzymes by hospital day four, with ALT levels rising to 426 U/L from a previously normal range at admission. In this case, switching the antibiotic resulted in a downtrend of ALT levels, suggesting that fluoroquinolones may also contribute to liver injury [7].

Additionally, a prospective study involving 679 subjects found that the average onset of drug-induced liver injury symptoms was four days after drug initiation. However, cases were reported as late as 39 days post-drug initiation, highlighting the importance of monitoring patients even after completing the medication course to detect delayed presentations of drug-induced liver injury [8].

A retrospective study identified risk factors for drug-induced liver injury (DILI) following Legionella infection. The study found that male sex (OR 6.975), elevated CRP (OR 1.182), and high hemoglobin levels (OR 1.640) before drug administration were predisposing factors for DILI [9]. In our case, the patient’s hemoglobin level was 13.1 g/dL, which was within normal limits. However, CRP was not measured before the initiation of antibiotics, limiting our ability to assess this risk factor.

*Legionella*, apart from infecting the lungs, can sometimes spread to other organs. The heart is the most frequent target beyond the lungs, but the kidneys and pancreas can also be affected. This ability to disseminate and cause systemic complications likely stems from the bacteria's capacity to cross the lung barrier and enter the bloodstream [10], [11], [12].

This case highlights the importance of vigilance regarding potential liver injury during Legionnaires' disease treatment. Clinicians must carefully balance the benefits of antibiotic therapy against its hepatotoxic risks and tailor treatment accordingly.

In conclusion, this case underscores the multifaceted nature of Legionnaires' disease. While *Legionella pneumophila* is primarily known for causing pneumonia, it can also lead to extrapulmonary complications such as acute hepatitis. Additionally, it highlights the challenge of drug-induced liver injury associated with antibiotics commonly used to treat Legionnaires' disease. Clinicians must maintain a high index of suspicion for Legionnaires' disease, particularly in the post-COVID era, and carefully weigh the risks and benefits of antibiotic therapy to ensure optimal patient outcomes.

## CRediT authorship contribution statement

**Sagar Modh:** Project administration, Writing – original draft, Writing – review & editing. **Mark Grijalva:** Project administration, Writing – original draft, Writing – review & editing. **Vignesh Krishnan:** Writing – original draft, Writing – review & editing. **Angel Chacko:** Writing – original draft, Writing – review & editing. **Shraboni Dey:** Writing – original draft, Writing – review & editing. **Paranjyothy Rao Pirangi Sanjeeva:** Writing – original draft, Writing – review & editing. **Adam Atoot:** Project administration, Writing – original draft, Writing – review & editing.

## Patient consent

A written Informed consent for the publication of this case report was obtained from the patient. To ensure patient confidentiality, we have omitted their name and any other details that could be used to identify them.

## Ethical approval

Received

## Funding

No funding received

## Declaration of Competing Interest

The authors declare that they have no known competing financial interests or personal relationships that could have appeared to influence the work reported in this paper.
